# Efficacy and safety of a 4-step versus a 5-step egg ladder in children with IgE-mediated hen's egg protein allergy: protocol for an open-label randomized controlled trial

**DOI:** 10.3389/falgy.2025.1658186

**Published:** 2025-09-15

**Authors:** Andrea Horvath, Anna Bujnowska, Agata Stróżyk, Maria Zemła, Anna Nowak-Węgrzyn, Katarzyna Grzela, Joanna Jerzyńska, Hania Szajewska

**Affiliations:** 1 Department of Paediatrics, Medical University of Warsaw, Warsaw, Poland; 2 Department of Paediatrics, Hassenfeld Children’s Hospital, New York University Grossman School of Medicine, New York City, NY, United States; 3 Department of Pediatrics, Gastroenterology and Nutrition, Collegium Medicum, University of Warmia and Mazury, Olsztyn, Poland; 4 Department of Pediatric Pneumonology and Allergy, Medical University of Warsaw, Warsaw, Poland; 5 Department of Pediatrics and Allergy, Korczak Pediatric Center, Medical University of Lodz, Lodz, Poland

**Keywords:** paediatrics, food allergy, egg ladder, IgE-mediated hen's egg allergy, children

## Abstract

**Background:**

Introducing baked egg into the diet of children with hen's egg allergy (HEA) has been shown to potentially accelerate the development of tolerance to non-heated egg. However, there is no standardized egg ladder (EL) protocol, and different scientific societies across countries recommend varying versions. This study aims to assess the efficacy and safety of the four-step EL (4-EL) compared with the five-step EL (5-EL) in children with IgE-mediated HEA.

**Methods:**

We will perform an open-label randomized trial with two-parallel arms in two departments if the same academic hospital. A total of 84 children with IgE-mediated HEA will be allocated in 1:1 ratio to introduce hen's egg allergy into their diet according to either 4-EL or 5-EL with 4-week break period between subsequent steps. Oral food challenge (OFC) with tested products at each subsequent step of the EL will be conducted in hospital settings. The primary outcome will be the percentage of children with tolerance to non-heated hen's egg proteins defined as non-allergic reaction to raw hen's egg (0.5–1 egg, depending on the age of the patient) during the last OFC; measured at the end of the 18-week observation period for the 4-EL and 24-week observation period for the 5-EL. Secondary outcomes will include the percentage of children with negative OFC to each EL step; the percentage of children with anaphylaxis (including the percentage of those who were treated with epinephrine); the percentage of children with exacerbation of atopic dermatitis; growth; compliance; and quality of life of the caregivers and parents anxiety about adverse events during their child's OFC.

**Discussion:**

This rigorously designed RCT will provide evidence on the efficacy and safety of the 4-EL in children with IgE-mediated HEA. The findings will inform guideline development groups and further confirmatory trials.

**Trial Registration Number:**

NCT07040111, date of registration: 27 June 2025.

## 1 Introduction

Hen's egg allergy (HEA) is one of the most common food allergies affecting the pediatric population, with an incidence of HEA of less than 1.2% in children under 2 years of age (1, 2). The standard of care for HEA is an elimination diet excluding hen's egg proteins. However, this approach, especially when followed long-term, presents several challenges, including delayed introduction of complementary foods, malnutrition, impaired growth, feeding difficulties and even social exclusion (3, 4).

Available evidence suggests that spontaneous resolution of HEA occurs in the majority of affected children over time. In the *EuroPrevall* study, 49% of children with HEA (*n* = 24/49) achieved tolerance to hen's egg proteins one year after diagnosis (1). Additionally, in the Australian *HealthNuts* cohort study, HEA resolved in 89% of participants (*n* = 287/323) by the age of 6 years (5). Nonetheless, a gradual reintroduction of heated egg using the egg ladder (EL) approach may accelerate the time to tolerance acquisition (6).

The egg ladder involves the gradual introduction of egg, beginning with less allergenic, extensively heated forms in small amounts, and progressing to lightly-cooked egg in regular, age-appropriate portions (7). The rationale for using the EL to assess tolerance acquisition is based on the lower allergenicity of baked compared to its unheated form. This reduction in allergenicity is attributed to the degradation of conformational IgE-binding epitopes caused by high-heat exposure (e.g., baking in the oven at 180°C for 30 min), as well as the “matrix effect” of the wheat-based batter – both shown to reduce IgE antibody binding to egg white proteins, thereby attenuating their allergenicity (8–10).

Leonard et al. demonstrated that the regular dietary inclusion of baked egg (e.g., a muffin or waffle containing 3.3 g of hen's egg proteins) reduced the time to tolerance induction to 50 months, compared to 78 months in children adhering to a strict egg avoidance diet (11). A 2025 systematic review summarized that in most studies, baked egg is safe and well-tolerated, with overall tolerance rates among children with HEA ranging from 70% to 97% (8, 9, 12). Furthermore, a 2024 systematic review and meta-analysis of 8 studies reported that 69% of children with HEA achieved tolerance to raw egg using the EL (95% CI: 0.5–0.85; *n* = 193) (8).

Allergology societies in different countries have proposed varying versions of the EL. However, discrepancies exist in both the number of steps included (ranging from 3 to 9 stages) and in the recommended intervals for progressing between each stage (ranging from 1 to 9 months) (12–17). Only one randomized controlled trial (RCT) has assessed the effect of a short-term EL protocol (18 months with 3–6 months break in periods) compared to a long-term protocol (30 months with a 9 months break in period) on hen's egg tolerance acquisition (12). This trial demonstrated a shorter time to achieve tolerance to unheated hen's egg (24 vs. 30 months) and a higher proportion of children attaining tolerance to raw egg (80% vs. 69%) in the short-term arm compared to control group (*n* = 78).

Currently, there are no standardized protocols for the assessment of tolerance acquisition using the EL approach. Furthermore, scientific evidence supporting the efficacy and safety of the EL in children with HEA remains limited.

## 2 Methods and analysis

The study design followed established reporting standards, including the 2025 CONSORT Consolidated extension (Consolidated Standards of Reporting Trials) (18) and the SPIRIT 2025 (Standard Protocol Items: Recommendations for Interventional Trials) guidelines (19). In addition, the design incorporated the COMFA core outcome set for IgE-mediated food allergy research (20).

### 2.1 Study objective and hypothesis

The primary aim of this study is to evaluate and compare the efficacy and safety of a 4-step egg ladder (4-EL) and a 5-step egg ladder (5-EL) in children diagnosed with IgE-mediated hen's egg protein allergy.

We hypothesize that the 4-EL, with 6-week intervals, will be sufficient for assessing tolerance to hen's egg proteins in children with IgE-mediated allergy and will result in a greater proportion of children achieving tolerance to raw egg white, confirmed by OFC, compared to the 5-EL, with the same timeframe. This corresponds to an 18-week tolerance acquisition period in the 4-EL group vs. 24 weeks in the 5-EL group.

There is some evidence suggesting that the introduction of baked egg in high-risk children (e.g., those with a history of anaphylaxis) may be both safe and effective (21). However, it remains unclear whether the initial tested dose of baked hen's egg protein in such cases should be lower than, or the same as, that recommended for the general pediatric HEA population.

Therefore, we decided to compare two ladder protocols: the 4-EL, beginning with a muffin containing 1.5 g of hen's egg proteins, and the 5-EL, in which the first two steps consist of muffins containing 0.75 g and 1.5 g of egg proteins, respectively. This approach allows for a reduced allergen load at initial exposure and a longer cumulative exposure to baked hen's egg proteins. Such a strategy may be more appropriate for specific subgroups of patients, particularly those considered to be at higher risk (21).

### 2.2 Study design

To compare both treatment strategies, we will conduct a multicenter, open-label, randomized superiority trial with two parallel arms and a 1:1 allocation ratio.

### 2.3 Study setting

Participants will be recruited, and the intervention will be implemented at two departments of the Medical University of Warsaw: the Department of Paediatrics and the Department of Paediatric Pneumonology and Allergology, as well as at the Department of Pediatrics and Allergy at the Medical University of Lodz (academic hospitals). All centers have substantial expertise in the management of pediatric food allergies, particularly in conducting oral food challenges (OFCs). In addition, the research team has extensive experience in the design and implementation of clinical trials. Participant recruitment is scheduled to begin in July 2025 and is expected to be completed within 36 months. Investigators will regularly review lists of potentially eligible patients with IgE-mediated HEA. Other team members will also be informed about study design and eligibility criteria to refer potential participants.

### 2.4 Eligibility criteria

Eligibility for study participation requires full compliance with all inclusion and exclusion criteria outlined in Table 1 at the time of enrollment.

**Table 1:** Inclusion and exclusion criteria for pediatric patients with hen's egg allergy.

Inclusion criteria	Exclusion criteria
Age ≥12 months and ≤5 years. Diagnosis of IgE-mediated HEA confirmed according to the EAACI guidelines by a positive OFC with hen's egg proteins. In children with high-risk HEA (e.g., history of anaphylaxis), diagnosis can be based on elevated specific IgE levels to hen's egg proteins and/or individual egg components and/or positive skin tests^a^. On a therapeutic elimination diet for at least 6 months, measured from the last allergic reaction to egg (in accordance with BSACI guidelines) (16). Eligible regardless of the risk of systemic reactions (e.g., anaphylaxis) or asthma. Good general health Written informed consent signed by the child's guardians. Demonstrated good cooperation from the patient's guardians.	Confirmed wheat allergy and/or celiac disease. Uncontrolled asthma, defined as the presence of shortness of breath, cough, chest tightness, or auscultatory changes despite treatment. Signs of exacerbation of a chronic disease. Signs of acute infectious disease (e.g., acute rhinitis, cough, subfebrile or febrile states). Exacerbation of another allergic disease (e.g., conjunctivitis, allergic rhinitis, atopic dermatitis). Anaphylaxis due to hen's egg proteins within the last 6 months. Use of antihistamines within 3–10 days before the challenge^b^ (depending on the drug and indication; time-limited contradiction – relative exclusion criterion). Acquired tolerance to baked hen's egg proteins or advancement to a higher step of the egg ladder. Use of immunosuppressive drugs or immunotherapy.

BSACI, British Society for Allergy and Clinical Immunology; EAACI, European Academy of Allergy and Clinical Immunology; HEA, Hen's egg allergy; HEPs, Hen's egg proteins; OFC, oral food challenge.

a Exceptions to the oral food challenge (OFC) protocol may be made for patients presenting with severe anaphylaxis or significantly elevated specific IgE levels (3.5 KU/L) to egg white proteins (designated as a high-risk challenge, where standard protocols contraindicate OFC due to anaphylaxis risk) (7, 21, 22).

b Temporal constraint (relative exclusion criterion).

Given that some studies have reported higher allergenicity of baked egg products prepared with non-wheat based batters compared to those with wheat-based batters, children with wheat allergy or celiac disease requiring wheat flour substitution will be excluded from participation (10).

### 2.5 Intervention

All OFCs will be conducted in accordance with the PRACTical ALLergy (PRACTALL) guidelines (7).

Participants with IgE-mediated HEA will be randomly assigned to one of two groups: the intervention group (4-EL) or the control group (5-EL), with both protocols conducted over 6-week intervals (Table 2 and Supplementary Table S1).

**Table 2:** Comparison of the 4-step (intervention) and 5-step (control) Egg ladder protocols in children with hen's Egg allergy.

Tested product (temperature, heating duration)	The amount of tested product	The amount of whole hen's egg proteins per portion [g]	The amount of hen's egg proteins per 100 g [g]	4- Step Egg Ladder (intervention group)	5- Step Egg Ladder (control group)
MUFFIN 0.75 Temp. 180°C (oven) Heating duration: 30 min (or until center is dry)	1 muffin (approx. 50 g)	0.75	1.5	–	STEP 1
6 weeks of regular dietary exposure to the tolerated quantity of the previously assessed forms					
MUFFIN 1.5 Temp. 180°C (oven) Heating duration: 30 min (or until center is dry)	1 muffin (approx. 50 g)	1.5	3	STEP 1	STEP 2
6 weeks of regular dietary exposure to the tolerated quantity of the previously assessed forms					
PANCAKE Temp. medium heat – approx. 180–190°C (frypan) Heating duration: 2–3 min both sides (until a golden-brown color is achieved)	1 pancake (approx. 84 g)	2.0	2.4	STEP 2	STEP 3
6 weeks of regular dietary exposure to the tolerated quantity of the previously assessed forms					
HARD- BOILED EGG Temp. approx. 100°C; should be added to the water once it reaches a rolling boil Heating duration: 10–12 min	1–3 years: 0.5–1 egg 4–5 years: 1 egg (approx. 50 g)	3.0 per 0.5 egg^a^ 6.0 per egg^a^	12.0	STEP 3	STEP 4
6 weeks of regular dietary exposure to the tolerated quantity of the previously assessed forms					
Almost raw egg: SOFT-BOILED EGG Temp. approx. 100°C; should be added to the water once it reaches a rolling boil Heating duration: 5 min	1–3 years: 0.5–1 egg 4–5 years: 1 egg approx. 50 g)	3.0 per 0.5 egg^a^ 6.0 per egg^a^	12.0	STEP 4	STEP 5
				Duration: 18 weeks	Duration: 24 weeks

a Amounts of hen's egg protein were estimated based on the average content in a medium egg (50 g).

The 4-EL protocol [muffin (1.5 g hen's egg proteins), pancake, hard-boiled egg, and soft-boiled egg] and the 5-EL protocol (beginning with a muffin containing 0.75 g of hen's egg proteins, followed by 1.5 g, and then the remaining steps of the 4-EL) are both based on modifications of previously published egg ladders used in clinical practice (13, 15).

Instead of using raw egg as the final step, we chose to assess tolerance to soft-boiled egg, which is considered almost raw. This decision is justified mainly by practical considerations to increase parents and child's acceptability, and to prioritize the reintroduction of foods that are more likely to be regularly consumed in everyday diets.

At each step of the ladder, a single food product was selected and applied consistently across both study arms. The quantity of hen's egg proteins at each stage was determined based on recommendations from the PRACTALL consensus report (7) and the BSACI guidelines (15).

#### 2.5.1 Supervised oral food challenge

Oral food challenges at each step of the egg ladder will be conducted under hospital conditions by a research physician. Data on the hen's egg protein content in the study products (per portion and per 100 g) are presented in Table 2.

Prior to each OFC, parents will receive detailed recipes for home preparation of the products, as well as information on medications that must be discontinued before the OFC (Supplementary Material 1). OFC will be performed after a minimum four-hour fast. For younger children, a light, low-fat snack (equivalent to 0.5 of a standard portion) may be allowed two hours prior to the scheduled challenge.

In accordance with PRACTALL consensus (7) the OFC will be conducted using a four-dose challenge protocol (1/16–1/8–1/4 – and the remaining amount of a muffin), with 30-minute intervals between each administered portion of the tested product. Following the final dose, the patient will be monitored in an inpatient setting for two hours. If no adverse reaction occurs, the child may be discharged and should be closely observed at home by a parent for an additional 24 h to rule out any delayed allergic reactions.

While the double-blind, placebo-controlled food challenge is considered the gold standard for the diagnosis of HEA (15), we will conduct open-label OFCs to enhance feasibility and encourage parental cooperation. To reduce bias, we will use the predetermined stopping criteria based on objective signs.

Despite providing standardized recipes and instructing parents to strictly follow the instructions, there is still a possibility of inaccurate food preparation (e.g., baking nine muffins instead of eight). Therefore, prior to each OFC, a researcher will weigh the test product (e.g., muffin or other food item) to precisely estimate the actual amount of hen's egg proteins consumed by the child. Both the tested portion and the total amount of the prepared egg-containing food will be weighed and recorded

#### 2.5.2 Duration of each ladder step and reintroduction of HEPs at home

The literature lacks a standardized egg ladder protocol, including the time intervals between its consecutive stages. Scientific societies suggest intervals ranging from 1 to 9 months (12–16). In the Canadian egg ladder protocol, a 1–3 month interval between stages is recommended (13). In this study, based on our own clinical experience, we hypothesized that a 6-week interval would be sufficient to assess the acquisition of tolerance to each subsequent stage of the egg ladder.

Following a negative OFC result and careful 24-h home observation, parents will be instructed to regularly reintroduce hen's egg proteins at home. This study follows the BSACI guidelines (15), which recommended the administration of hen's egg proteins three times per week. Over a six-week period, caregivers will introduce hen's egg proteins in the same quantity and form that did not elicit an allergic reaction during the preceding OFC.

Participants progressing to advanced EL stages will incorporate into their diet the dose of hen's egg proteins tolerated during the previous OFC, along with one to two additional portions of hen's egg proteins products from lower EL steps. The total intake should not exceed three hen's egg proteins-containing products per day. To ensure safety, home consumption of higher-step EL products is not permitted until tolerance has been confirmed through a hospital-based OFC.

Tested food products and their amount were developed according to the BSACI (15) and PRACTALL guidelines (7) and RCT by de Vlieger et al. (12) (Table 2).

#### 2.5.3 Children with a positive OFC

A positive OFC is defined according to the criteria outlined in the PRACTALL guidelines (Supplementary Table S2) (7). The OFC will be stopped if any red ('Stop') criterion is met, or if two (2) orange (“Observation”) criteria are met.

In the event of a positive OFC result with baked egg (Step 1 of the EL), children will continue with an elimination diet.

A positive OFC result at later stages of the ladder will prevent progression to the next step. At home, the allergen will continue to be administered in the previously tolerated form, with dosage and frequency determined by the research team.

All children with a positive OFC will be excluded from further participation in the study and will receive continued care from the department in accordance with established clinical standards. A repeat OFC will be scheduled within 6–12 months, based on an individual risk assessment of potential adverse events and the attending physician's clinical judgment.

### 2.6 Study procedure

Children aged 12 months to 5 years with a confirmed diagnosis of IgE-mediated HEA will be invited to participate in the study. During a medical visit at the department, the recruiting physician will conduct a thorough interview with the parent or guardian, perform a physical examination of the child, review the patient's previous medical records – and perform the eligibility screening.

Children who meet the study eligibility criteria will be randomly assigned to either the intervention or control group. Parents will receive detailed information regarding the study procedure, the upcoming OFC, and the potential benefits and risks of participation. Informed consent will then be obtained from each child's legal guardian, with the form signed in duplicate.

All participating children will begin the introduction of hen's egg proteins into their diet following either the 4-EL (intervention group) or 5-EL (control group). Each subsequent stage of the EL will be initiated with an OFC involving progressively less processed forms of hen's egg proteins: muffin with 0.75 g; muffin with 1.5 g; pancake; hard-boiled egg; soft-boiled egg (Table 2). During the 6-week interval between each stage, parents will introduce the allergen at the previously tolerated dose in the home setting (see Intervention).

#### 2.6.1 Parental education on allergen introduction in the home setting

The study products should be administered at home, preferably the early afternoon, after the child returns from daycare, to allow for optimal monitoring of any delayed reactions. Parents will be encouraged to keep a minimum interval of 2 h between servings. If the allergen is not administered for more than a week, the interval between subsequent OFCs should be extended proportionally to the length of the break.

The study product must not be administered on an empty stomach. In the presence of acute infectious symptoms, administration of the scheduled portion of hen's egg proteins should be postponed for at least three days or until symptoms resolve and child is back to baseline, because as a cofactor it may affect the child's tolerance to ingested allergens (23).

Throughout the intervention phase, a strict elimination of hen's egg proteins outside the prescribed product must be maintained. The use of commercially available food products containing hen's egg proteins – including those listing them as ingredients or potentially containing trace amounts – is not recommended due to the unknown hen's egg proteins content, in order to prevent unintentional cumulative allergen exposure.

To ensure participant safety, all parents will receive both verbal and written information concerning the potential for adverse reactions, including anaphylaxis, during home-based allergen administration. This will be accompanied by detailed management protocols. Prior to initiating the home introduction of the study food, study personnel will verify that all required emergency medications are available, as specified in the adverse event protocol. In addition, parents and caregivers will undergo comprehensive training on the recognition and appropriate management of allergic reactions.

Furthermore, parents are encouraged to contact the research team for guidance if any difficulties arise during progression through the egg ladder. It is emphasized that withdrawal of consent at any stage of the study will in no way affect the child's continued medical care or access to appropriate treatment.

#### 2.6.2 Assessments

Anthropometric data for the child, specifically height and weight, will be collected at study enrollment and at the end of the observation period (either at 18 or 24 weeks, depending on the assigned study arm). Weight (in kilograms) and standing height (in centimeters) will be measured according to standardized protocols.

Prior to the initial OFC and following completion of the egg ladder, the subjective quality of life of all enrolled children and their caregivers will be assessed using the validated, allergy-specific Parent Form of the Food Allergy Quality of Life Questionnaire (FAQLQ-PF) (24). The FAQLQ-PF was developed to measure parental perceptions of health-related quality of life in children aged 0–12 years with food allergies and includes three domains: emotional impact, food anxiety, and social and dietary limitations. In this study, only the age-appropriate versions of the questionnaire for children under 4 years and for those aged 4–6 years will be used, in accordance with the inclusion criteria. These versions contain 14 and 26 items, respectively. A total score ranging from 0 to 6 is calculated as the mean of the subscale scores.

Prior to each OFC, caregivers will assess their level of anxiety specifically related to the OFC using the Subjective Units of Distress Scale (SUDS) (25). On this scale, parents will rate their anxiety from 1 to 100, where 0 represents no anxiety or complete relaxation, and 100 represents extreme anxiety – the highest level of distress ever experienced.

In children with atopic dermatitis, objective skin lesions will be evaluated using the objective SCORAD index (oSCORAD) (26), a validated variant of the Severity Scoring of Atopic Dermatitis (SCORAD). Assessment will be performed prior to the initial OFC and during follow-up examinations at the end of each observation period.

The SCORAD index is a key tool for evaluating the severity of atopic dermatitis and monitoring treatment response It includes both objective measures (extent and intensity of skin lesions), and subjective symptoms (pruritus and sleep disturbance) reported over the preceding 72 h. The oSCORAD omits the subjective component, focusing exclusively on objective clinical findings. The maximum oSCORAD score is 83, with an optional addition of up to 10 points for disfiguring or function-limiting changes in severe cases. Severity categories are defined as follows: mild (<15), moderate (15–40), and severe (>40).

All children who experience anaphylaxis during the study period (either over 18 or 24 weeks, depending on the assigned study arm) will be assessed using the updated 2023 World Allergy Organization (WAO) clinical criteria for anaphylaxis (27). This scale categorizes allergic reactions during allergen exposure into five grades, with anaphylaxis defined as reactions falling within grades 3–5.

#### 2.6.3 Laboratory analysis

In all participants, blood samples will be collected prior to each oral food challenge to measure total IgE (tIgE) and specific IgE (sIgE) antibodies to hen's egg white and ovomucoid (Gal d 1). Previous studies have demonstrated the value of assessing the sIgE/tIgE ratio as a predictor of OFC outcomes (28, 29).

In addition, a basophil activation test (BAT) will be performed prior to each OFC. As a functional assay, BAT has demonstrated greater accuracy than sIgE measurement alone in identifying baked and lightly cooked egg allergy. Krawiec et al. (29) found that BAT improves the predictive value for a positive OFC outcome with baked egg and reduces the number of OFCs in children aged 6 months to 15 years with suspected egg allergy.

Both sIgE/tIgE ratio and BAT may support the identification of children who are more likely to tolerate food allergens during OFC. However, its role in predicting response to OFC with baked egg remains to be fully established.

### 2.7 Adherence

To systematically assess participant adherence and monitor the occurrence of anaphylactic events, parents will be instructed to meticulously document, on a daily basis, the quantity and frequency of at-home allergen administration during the final week preceding each OFC, as well as to record any suspected adverse reactions occurring at any time throughout the six-week interval between OFCs, using the Egg Ladder Monitoring Diary (developed by the study investigators, Additional File 2*****). This diary in paper form will be maintained throughout the six-week intervals between OFCs.

### 2.8 Follow-up

Patients will be followed up by the research team to assess their ongoing tolerance and continued inclusion of egg in their diet at 6 weeks after completing the EL.

### 2.9 Sample size justification

This study is designed as a randomized controlled trial (RCT) to compare the clinical effectiveness and safety of two versions of an egg ladder protocol: one with four steps and the other with five steps, in children with IgE-mediated hen's egg allergy.

A formal sample size calculation based on statistical power was not feasible due to the lack of standardized data regarding allergen content and clinical reactivity across different baked egg preparations used in oral food challenges. As noted in the existing literature, studies assessing the immunological and clinical progression through the egg ladder remain limited, and standardized endpoints have not yet been universally defined.

Therefore, this trial was informed by available clinical evidence and precedent from similar studies involving the milk ladder in cow's milk-allergic children. In particular, a comparable RCT evaluating the impact of introducing baked cow's milk on tolerance development included 84 children (42 per arm) and demonstrated sufficient power to detect clinically meaningful differences (14).

Moreover, a recent randomized trial by De Vlieger et al. (12), which evaluated short- vs. long-term guided gradual egg reintroduction in baked egg–tolerant children, also enrolled 78 children (39 per arm) and successfully identified significant differences in tolerance acquisition timelines.

Based on this evidence and our clinical experience, a total of 94 children (47 per group) will be recruited to allow for an anticipated 10% dropout rate, ensuring that at least 84 participants (42 per group) complete the trial. This sample size is considered both feasible and appropriate to meet the study objectives while allowing an initial exploration of clinical differences between the two egg ladder variants. This pilot trial will generate effect size estimates to inform future confirmatory studies and formal sample size calculations.

### 2.10 Study duration and rationale

Based on evidence from the literature (30), a study duration of 36 months is considered sufficient to complete participant recruitment and implement the interventions across both study subpopulations.

### 2.11 Outcomes

#### 2.11.1 Primary outcome

The primary outcome is the percentage of children who acquire tolerance to soft-boiled hen's egg (almost raw hen's egg proteins), as determined by a negative OFC at the final step of the EL. Soft-boiled egg will be administered at the end of the observation period (18 or 24 weeks, depending on the assigned study arm).

The challenge dose will consist of 0.5–1 egg for children aged 1–3 years and 1 egg for those aged 4–5 years, corresponding to a maximum total of 6 g of hen's egg proteins. The outcome will be reported as a difference between two study arms.

#### 2.11.2 Secondary outcomes

All secondary outcomes will be reported as a difference between two study arms.

- The percentage of children with a negative OFC at each step of the EL protocol upon completion of the study's observation period (18 weeks and 24 weeks, depending on the study arm), calculated as the proportion of children tolerating the full planned dose for each step.
- The proportion of children who experience anaphylaxis during the observation period (18 or 24 weeks, depending on study arm), defined as reactions with severity grades 3–5 according to the updated 2023 WAO clinical criteria for anaphylaxis (27).
- The proportion of children with anaphylaxis (grades 3–5, per 2023 WAO criteria) who required epinephrine administration, categorized by:
  ○ one or two doses of intramuscular epinephrine with a good clinical response (less severe reaction, grade 3),
  ○ more than 2 doses of intramuscular epinephrine,
  ○ lack of significant clinical improvement after two doses (severe reactions, grades 4–5),
  ○ measured during the observation period (18 or 24 weeks, depending on the study arm).
- The proportion of children who experienced systemic allergic reactions (SAR) of grades 1, 2, and 3 according to the 2023 WAO grading system, with specific reference to gastrointestinal symptoms:
  ○ grade 1: includes nausea and/or mild abdominal pain without altered activity;
  ○ grade 2: persisting (≥20 min) and non-distractable abdominal pain and/or vomiting (not due to gag or taste aversion) and/or diarrhea;
  ○ grade 3: severe, persistent (≥20 min), intractable abdominal pain and/or vomiting (excluding retching or taste aversion) and/or diarrhea, assessed during each OFC measured during the observation period (18 or 24 weeks, depending on the study arm).
- In the subgroup of children with AD, changes in the total score of the oSCORAD index (26) will be assessed before and after each OFC. Any documented exacerbation of AD symptoms following OFC will be also recorded.
- Change in age-adjusted *z*-scores for weight and length/height, evaluated using the WHO growth charts (31), from baseline (prior to the first OFC) to the end of the observation period (18 or 24 weeks, depending on the study arm).
- Change in the total score of the FAQLQ-PF (24), at the end of the observation period (18 weeks and 24 weeks, depending on the study arm) from baseline.
- Mean difference in caregiver anxiety levels, measured by the Subjective Units of Distress Scale (SUDS) (25) prior to each OFC, between study groups at each time point.
- The proportion of children with full adherence to the intervention protocol, defined as consumption of at least one portion of a tolerated hen's egg proteins dose three times during last week of break in period, in the same form and quantity as tested during the OFC. Data will be collected through the Egg Ladder Monitoring Diary and assessed throughout each break in period (between the following steps of each ladder).

#### 2.11.3 Exploratory outcomes

Sensitivity, specificity, and positive predictive value of the sIgE/tIgE ratio and basophil activation test (BAT) in predicting positive OFC outcomes for each form of hen's egg will be also assessed.

### 2.12 Concomitant care

If a participant is suspected of having a food allergy other than HEA, appropriate diagnostic and management procedures will be implemented. However, to preserve the internal validity of the study, any concurrent interventions that could potentially confound study outcomes, such as immunotherapy, will be contraindicated during the study period.

Parents will be instructed to promptly contact the study physician if any health concerns arise.

### 2.13 Study discontinuation criteria

Participant withdrawal from the study may occur under the following circumstances: voluntary withdrawal of consent by one or both parents/legal guardians, any perceived risk to the child's health, non-adherence to the study protocol, loss of contact with the participant, or the formal conclusion of the study.

In the event of premature withdrawal, the investigator will ensure that all evaluable endpoints are documented in the Clinical Research Form (CRF), unless the parents/legal guardians explicitly refuse further participation. If the discontinuation of the intervention is due to a confirmed and/or potential adverse event, the child will continue to be monitored, with all relevant observations recorded in both the medical records and the CRF.

### 2.14 Study modifications

In the event of adverse events occurring during an oral food challenge (OFC), the procedure will be immediately discontinued, and appropriate medical management will be initiated based on the child's clinical presentation, in accordance with the department's standard protocols. The interval between subsequent steps of the egg ladder will be determined on an individual basis.

If an adverse reaction occurs following the consumption of a tested food item at home, the child will be examined by the attending physician to determine the appropriate course of action.

In case of substantial feeding difficulties, different form of tested form may be proposed to a child. However, the same amount of hen's egg proteins per serving and temperature and heating settings must be maintained (e.g., crepes instead of pancakes; hardly cooked scrambled eggs instead of hard-boiled egg).

### 2.15 Harms

This study is of a cognitive-practical nature, and participants may not derive any direct clinical benefit. The proposed OFC procedures at each stage of hen's egg protein tolerance assessment are consistent with standard clinical practice. The only distinction introduced by this study is the random allocation to either an 18-week or 24-week observation arm. Outside the context of the study, the timing between OFCs would be determined solely at the discretion of the child's attending physician.

The assessment of patient and caregiver quality of life, as well as caregiver anxiety levels, requires an additional time commitment of approximately 15 min to complete standardized questionnaires designed to assess these domains.

All adverse events will be systematically documented in the Clinical Research Form (CRF) and categorized based on the International Council on Harmonization Good Clinical Practice (ICH-GCP) guidelines (32). Each event will be thoroughly evaluated to determine its duration, severity, and potential relationship with the administered intervention.

To ensure timely reporting and clinical assessment of any adverse events, participants will have access to the principal investigator via telephone and electronic mail.

In accordance with the CONSORT extension for harms reporting (33), all reported symptoms will be documented for all randomized participants, regardless of whether they complete the study. Adverse event data will be reported separately for each study arm and for each specific type of event, including the exact frequency of occurrence and distinguishing between participants with single and recurrent events.

### 2.16 Sequence generation and allocation concealment

Children will be randomised to either the intervention or control group immediately after enrolment. An independent investigator with no clinical involvement in the trial will generate random assignments using a computer-generated random number table. The random number will be generated in blocked randomization (using the block of 2) with a 1:1 allocation ratio. The randomization will be created using StatsDirect statistical software (Stats-Direct statistical software; http://www.statsdirect.com; England: StatsDirect, 2024).

Allocation concealment will be ensured through the use of opaque, sealed and numbered envelopes. The data regarding intervention assignments will be stored in a sealed envelope in a secure location within the administrative part of the department. An independent person will open the next consecutively numbered envelope and provide the allocation information to the study physician.

Since this is an open-label trial, both the participants and the study team will be aware of the treatment allocation on randomization. In the event of any medical issues, the assigned treatment information will already be accessible to the study physicians and principal researcher, eliminating the need for code-breaking procedures.

### 2.17 Blinding

Due to the nature of the intervention, this study will be unblinded. Participants, caregivers, investigators and outcome assessors will all be aware of treatment allocation.

### 2.18 Data collection and management

All study participants will be assigned a unique study identification number. CRFs will be completed on paper forms. Data will then be entered and stored in a password-protected electronic database. The original paper copies of CRFs and all study data will be stored in a locker within the study site, accessible to the involved researchers only.

### 2.19 Statistical analysis

Descriptive statistics will be used to summarize baseline characteristics. For continuous variables, comparisons between groups will be made using the Student's *t*-test, Mann–Whitney *U*-test or Wilcoxon signed rank test, depending on whether or not the variables are normally distributed. The appropriate statistical test will be employed to compare percentages, either the χ^2^ test or Fisher's exact test.

For continuous outcomes, the differences in means or medians (depending on the distribution of data), and for dichotomous outcomes, the relative risk (RR) and number needed to treat (NNT), all accompanied by a 95% CI, will be calculated. The difference between study groups will be considered statistically significant if the *p*-value is less than 0.05, if the 95% CI for RR does not include 1.0, or if the 95% CI for mean difference does not include 0. All statistical tests will be two-tailed and performed at the 5% level of significance.

An intention-to-treat (ITT) analysis including all randomised participants, regardless of protocol adherence or withdrawal, will be applied. A per-protocol analysis that includes all participants who finish the study according to the protocol will also be performed.

The number of missing data with reasons will be reported for each study arm, and compared qualitatively between groups, if feasible. Multiple imputation methods for missing data will be applied to address missing data, as recommended (34).

### 2.20 Monitoring

The study will be carried out in accordance with the protocol, as it will be registered. No changes in the study protocol are expected to be made after the study starts. However, in case of any unexpected circumstances requiring alterations of the protocol, changes will be immediately applied to the protocol registry site at https://www.clinicaltrials.gov, and, if relevant enough, reported to the Bioethics committee of each participating institution. An independent Data and Safety Monitoring Board (DSMB) will be created before the start of the study. The DSMB will review data after recruitment from 25%, 50% and 75% of participants to assess the study progress (including rate of recruitment, completeness of data and their appropriate collection) and all the adverse events. The number of recruited patients will be monitored and kept up to date; appropriate changes (i.e., training of the recruiting physicians, study leaflets, addition of new recruitment centres) will be applied to the study procedure and protocol if the pace of recruitment is not high enough (or not sufficient) to finish the study within the established time of 4 years.

## 3 Discussion

Currently, there are no standardized protocols for the assessment of tolerance acquisition using the EL approach. Furthermore, scientific evidence supporting the efficacy and safety of the EL in children with HEA remains limited. According to the BSACI guidelines, reintroduction of baked egg as an ingredient may be performed in children aged 12 months or 6 months from the last reaction (15). Additionally, regular ingestion of baked egg may accelerate the acquisition of tolerance to unheated egg and reduce dietary exclusions.

This study will assess the efficacy of the 4-EL compared to the 5-EL in children with confirmed IgE-mediated HEA. Due to limited evidence, high-quality trials are needed. However, no study is perfect, and this trial also has some limitations. The risk of bias is associated with the lack of blinding, which is not feasibility due to the nature of the intervention. Although participant will receive the intervention in both study arms (the 4-EL or 5-EL), some parents may prefer a different ladder than the one assigned (i.e., the shorter arm may be associated with fewer hospital visits). A second limitation is the home preparation of tested foods by caregivers. Although standardized recipes will be provided, a risk of inaccurate food preparation or modification cannot be excluded. To minimize this bias, caregivers will be asked before each challenge, whether any modifications were made, and the total amount and portion of the prepared foods will be weighed. Another limitation is the absence of a formal sample size calculation and the pilot nature of this trial. Due to limited evidence, the sample size was only estimated. However, it is considered sufficient to detect a clinical difference between these two egg ladders and to inform further confirmatory trials.

This rigorously designed RCT will help to compare the efficacy of two ladders: the 4-EL which begins with a higher amount of baked hen's egg proteins (1.5 g), and the 5-EL, in which gradual reintroduction starts with two steps – muffins containing 0.75 g and 1.5 g of egg proteins, respectively. Currently, it is unclear whether the initial reintroduced dose of baked hen's egg protein in children with IgE-mediated HEA, especially those in high-risk groups, should differ from that used in children with mild and moderate non-IgE mediated HEA. This study will be conducted by a research center experienced in RCTs involving children and food allergy. The study team is supported by ANW, who serves as a scientific mentor and provides expert oversight in protocol development and clinical decision-making in complex cases. The findings of this RCT will address current evidence gaps and inform guideline development groups regarding the efficacy and safety of the 4-EL in children with IgE-mediated HEA.

## References

[1] Xepapadaki P Fiocchi A Grabenhenrich L Roberts G Grimshaw KE Fiandor A Incidence and natural history of hen’s egg allergy in the first 2 years of life-the EuroPrevall birth cohort study. Allergy. (2016) 71(3):350–7. 10.1111/all.12801 26514330

[2] Grimshaw KEC Roberts G Selby A Reich A Butiene I Clausen M Risk factors for hen’s egg allergy in Europe: EuroPrevall birth cohort. J Allergy Clin Immunol Pract. (2020) 8(4):1341–8.e5. 10.1016/j.jaip.2019.11.040 31846795

[3] Meyer R De Koker C Dziubak R Venter C Dominguez-Ortega G Cutts R Malnutrition in children with food allergies in the UK. J Hum Nutr Diet. (2014) 27(3):227–35. 10.1111/jhn.12149 23937486

[4] Warren CM Jiang J Gupta RS. Epidemiology and burden of food allergy. Curr Allergy Asthma Rep. (2020) 20(2):6. 10.1007/s11882-020-0898-7 32067114 PMC7883751

[5] Peters RL Guarnieri I Tang MLK Lowe AJ Dharmage SC Perrett KP The natural history of peanut and egg allergy in children up to age 6 years in the HealthNuts population-based longitudinal study. J Allergy Clin Immunol. (2022) 150(3):657–65.e13. 10.1016/j.jaci.2022.04.008 35597613

[6] Bloom KA Huang FR Bencharitiwong R Bardina L Ross A Sampson HA Effect of heat treatment on milk and egg proteins allergenicity. Pediatr Allergy Immunol. (2014) 25(8):740–6. 10.1111/pai.12283 25251921

[7] Sampson HA Arasi S Bahnson HT Ballmer-Weber B Beyer K Bindslev-Jensen C AAAAI-EAACI PRACTALL: standardizing oral food challenges-2024 update. Pediatr Allergy Immunol. (2024) 35(11):e14276. 10.1111/pai.14276 39560049

[8] Anagnostou A Mack DP Johannes S Shaker M Abrams EM DeSanto K The safety and efficacy of baked egg and milk dietary advancement therapy: a systematic review and meta-analysis. J Allergy Clin Immunol Pract. (2024) 12(9):2468–80. 10.1016/j.jaip.2024.06.016 38901613

[9] Gallagher A Delgado Mainar P Cronin C Muñoz C Calleja JR Loughnane C Managing egg allergy: a systematic review of traditional allergen avoidance methods and emerging graded exposure strategies. Pediatr Allergy Immunol. (2025) 36(4):e70075. 10.1111/pai.70075 40167149 PMC11960040

[10] Gonzalez PM Cassin AM Durban R Upton JEM. Effects of food processing on allergenicity. Curr Allergy Asthma Rep. (2025) 25(1):9. 10.1007/s11882-024-01191-5 39804418

[11] Leonard SA Sampson HA Sicherer SH Noone S Moshier EL Godbold J Dietary baked egg accelerates resolution of egg allergy in children. J Allergy Clin Immunol. (2012) 130(2):473–80.e1. 10.1016/j.jaci.2012.06.006 22846751 PMC3428057

[12] De Vlieger L Nuyttens L Matton C Diels M Verelst S Leus J Guided gradual egg-tolerance induction in hen’s egg allergic children tolerating baked egg: a prospective randomized trial. Front Allergy. (2022) 3:886094. 10.3389/falgy.2022.886094 35769568 PMC9234941

[13] Chomyn A Chan ES Yeung J Vander Leek TK Williams BA Soller L Canadian food ladders for dietary advancement in children with IgE-mediated allergy to milk and/or egg. Allergy Asthma Clin Immunol. (2021) 17(1):83. 10.1186/s13223-021-00583-w 34353372 PMC8340453

[14] Gotesdyner L Zeldin Y Machnes Maayan D Efron A Stauber T Maoz Segal R A structured graduated protocol with heat denatured eggs in the treatment of egg allergy. Pediatr Allergy Immunol. (2019) 30(8):824–32. 10.1111/pai.13115 31419328

[15] Leech SC Ewan PW Skypala IJ Brathwaite N Erlewyn-Lajeunesse M Heath S BSACI 2021 guideline for the management of egg allergy. Clin Exp Allergy. (2021) 51(10):1262–78. 10.1111/cea.14009 34586690

[16] Venter C Meyer R Ebisawa M Athanasopoulou P Mack DP. Food allergen ladders: a need for standardization. Pediatr Allergy Immunol. (2022) 33(1):e13714. 10.1111/pai.13714 34882843

[17] Cotter S Lad D Byrne A Hourihane JO. Home-based graded exposure to egg to treat egg allergy. Clin Transl Allergy. (2021) 11(8):e12068. 10.1002/clt2.12068 34667590 PMC8506942

[18] Hopewell S Chan AW Collins GS Hróbjartsson A Moher D Schulz KF CONSORT 2025 statement: updated guideline for reporting randomized trials. JAMA. (2025) 31(6):1776–83. 10.1001/jama.2025.4347 40229553

[19] Chan A-W Boutron I Hopewell S Moher D Schulz KF Collins GS SPIRIT 2025 statement: updated guideline for protocols of randomized trials. Nat Med. (2025) 31(6):1784–92. 10.1038/s41591-025-03668-w 40295741

[20] Demidova A Drewitz KP Kimkool P Banjanin N Barzylovich V Botjes E Core outcome set for IgE-mediated food allergy clinical trials and observational studies of interventions: international Delphi consensus study “COMFA”. Allergy. (2024) 79(4):977–89. 10.1111/all.16023 38433402

[21] Gallagher A Cronin C Heng TA McKiernan A Tobin C Flores L Dietary advancement therapy using milk and egg ladders among children with a history of anaphylaxis. J Allergy Clin Immunol Pract. (2024) 12(8):2135–43. 10.1016/j.jaip.2024.04.057 38729302

[22] Santos AF Riggioni C Agache I Akdis CA Akdis M Alvarez-Perea A EAACI guidelines on the diagnosis of IgE-mediated food allergy. Allergy. (2023) 78(12):3057–76. 10.1111/all.15902 37815205

[23] Christensen MJ Eller E Kjaer HF Broesby-Olsen S Mortz CG Bindslev-Jensen C. Exercise-induced anaphylaxis: causes, consequences, and management recommendations. Expert Rev Clin Immunol. (2019) 15(3):265–73. 10.1080/1744666X.2019.1562904 30601082

[24] DunnGalvin A Koman E Raver E Frome H Adams M Keena A An examination of the food allergy quality of life questionnaire performance in a countrywide American sample of children: cross-cultural differences in age and impact in the United States and Europe. J Allergy Clin Immunol Pract. (2017) 5(2):363–8.e2. 10.1016/j.jaip.2016.09.049 28017626

[25] Joshi SP Wong AI Brucker A Ardito TA Chow SC Vaishnavi S Efficacy of transcendental meditation to reduce stress among health care workers: a randomized clinical trial. JAMA Netw Open. (2022) 5(9):e2231917. 10.1001/jamanetworkopen.2022.31917 36121655 PMC9486450

[26] Chopra R Vakharia PP Sacotte R Patel N Immaneni S White T Severity strata for eczema area and severity Index (EASI), modified EASI, scoring atopic dermatitis (SCORAD), objective SCORAD, atopic dermatitis severity Index and body surface area in adolescents and adults with atopic dermatitis. Br J Dermatol. (2017) 177(5):1316–21. 10.1111/bjd.15641 28485036

[27] Turner PJ Ansotegui IJ Campbell DE Cardona V Carr S Custovic A Updated grading system for systemic allergic reactions: joint statement of the world allergy organization anaphylaxis committee and allergen immunotherapy committee. World Allergy Organ J. (2024) 17(3):100876. 10.1016/j.waojou.2024.100876 38361745 PMC10867340

[28] Esty B Maciag MC Bartnikas LM Petty CR MacGinnitie AJ Sheehan WJ Predicting outcomes of baked egg and baked milk oral food challenges by using a ratio of food-specific IgE to total IgE. J Allergy Clin Immunol Pract. (2021) 9(4):1750–2.e1. 10.1016/j.jaip.2020.11.004 33217611 PMC8043965

[29] Gupta RS Lau CH Hamilton RG Donnell A Newhall KK. Predicting outcomes of oral food challenges by using the allergen-specific IgE-total IgE ratio. J Allergy Clin Immunol Pract. (2014) 2(3):300–5. 10.1016/j.jaip.2013.12.006 24811021

[30] Esmaeilzadeh H Alyasin S Haghighat M Nabavizadeh H Esmaeilzadeh E Mosavat F. The effect of baked milk on accelerating unheated cow’s milk tolerance: a control randomized clinical trial. Pediatr Allergy Immunol. (2018) 29(7):747–53. 10.1111/pai.12958 30027590

[31] WHO Multicentre Growth Reference Study Group. WHO child growth standards based on length/height, weight and age. Acta Paediatr Suppl. (2006) 450:76–85. 10.1111/j.1651-2227.2006.tb02378.x 16817681

[32] Dixon JR Jr. The international conference on harmonization good clinical practice guideline. Qual Assur. (1998) 6(2):65–74. 10.1080/105294199277860 10386329

[33] Butcher NJ Monsour A Mew EJ Chan AW Moher D Mayo-Wilson E Guidelines for reporting outcomes in trial reports: the CONSORT-outcomes 2022 extension. JAMA. (2022) 328(22):2252–64. 10.1001/jama.2022.21022 36511921

[34] Jakobsen JC Gluud C Wetterslev J Winkel P. When and how should multiple imputation be used for handling missing data in randomised clinical trials – a practical guide with flowcharts. BMC Med Res Methodol. (2017) 17(1):162. 10.1186/s12874-017-0442-1 29207961 PMC5717805

