# Supplementary material for: Efficacy and safety of a 4-step versus a 5-step egg ladder in children with IgE-mediated hen's egg protein allergy: protocol for an open-label randomized controlled trial
Source: Front Allergy. 2025 Sep 15;6:1658186. doi: 10.3389/falgy.2025.1658186 (PMC12477030; doi:10.3389/falgy.2025.1658186)
Supplement: Supplementary file 1 [file Datasheet1.docx]

**SUPPLEMENTARY TABLES**

**Supplementary Table 1. Study Timeline**

|  | **Trial period** | | | | | |
| --- | --- | --- | --- | --- | --- | --- |
|  | **Enrollment** | **Post randomization** | | | **Close-out** | |
|  |  | **1-STEP OFC** | **2-STEP OFC** | **3-STEP OFC** | **4-STEP OFC** | **5-STEP OFC** |
| **TIMEPOINT** | **-t1 to 0** | **1 day** | **6th week** | **12th week** | **18th week** | **24th week** |
| Enrollment | X |  |  |  |  |  |
| Eligibility screen | X |  |  |  |  |  |
| Informed consent | X |  |  |  |  |  |
| Allocation and randomization | X |  |  |  |  |  |
| **INTERVENTION** | | | | | | |
| 4-step EL (intervention) |  | X | X | X | X |  |
| 5-step EL (comparator) |  | X | X | X | X | X |
| **ASSESSMENTS** | | | | | | |
| Hen’s egg specific-IgE | X |  |  |  |  |  |
| Skin Prick Test | X |  |  |  |  |  |
| BAT | X |  |  |  |  |  |
| oSCORAD |  | X | X | X | X | X |
| SUDS |  | X | X | X | X | X |
| FAQLQ-PF |  | X | X | X | X | X |
| Anthropometry |  | X | X | X | X | X |
| Risk of anapylaxis (WAO scale) |  |  |  |  |  |  |
| Diary |  |  |  |  |  |  |
| Adverse events |  |  |  |  |  |  |

BAT, basophil activation test; EL, egg ladder; FAQLQ-PF, Food Allergy Quality of Life Questionnaire; oSCORAD, objective Severity Scoring of Atopic Dermatitis; SUDS, Subjective Units of Distress Scale; WAO, World Allergy Organization

**Supplementary Table 2. Definition of a Positive OFC According to PRACTALL consensus (7)**

| **Symptom** | **Proceed (green)** | **Pause and reassess (orange)** | **Stop challenge (red)** |  |
| --- | --- | --- | --- | --- |
| **Rash: erythema** | Few areas of faint erythema | <50% of body surface | Generalized erythema (>50% body surface) |  |
| **Rash: urticaria** | Limited to perioral region or due to contact | 1–2 lesions (not perioral or due to contact) | At least 3 lesions (not periorial or due to contact) | Local skin reactions due to contact (including lip contact with challenge dose) excluded |
| **Angioedema** | Prominent lip or ear edema | Facial edema including new-onset uvula edema | Generalized edema | Facial (including periocular) swelling should be prominent and not due to local rubbing or crying. If crying/rubbing causes local swelling, consider delaying the next FC dose to see if other symptoms develop |
| **Pruritus** | Scratching any area |  |  | Not considered a stopping criterion |
| **Eyes** | Minimal reddening, rubbing of eyes | Conjunctival hyperemia without prior rubbing |  | Periocular rubbing or crying is a common cause of conjunctival reddening |
| **Nasal symptoms** | Mild, infrequent rhinitis | Persistent significant rhinorrhea/sneezing |  | Note mild nasal symptoms are common during FC and therefore a poor indicator of objective reaction |
| **Cough** | Intermittent cough associated with throat clearing | Frequent cough without respiratory compromise | Cough associated with respiratory compromise (anaphylaxis) | If cough is present without evidence of respiratory compromise (e.g., significant tachypnoea, fall in oxygen saturations, use of accessory muscles, wheezing, PEFR decrease >20% with good technique), consider whether to terminate the FC (which could lead to an equivocal result if no other symptoms develop) or adopt “watchful waiting” (and delay the next FC dose) |
| **Wheezing** |  |  | Any wheezing (anaphylaxis) | Reduced air entry or “added sounds” on auscultation may precede wheeze |
| **Chest tightness** | Isolated chest tightness without |  | Chest tightness with PEFR fall >20% (good technique) (anaphylaxis) | Chest tightness is subjective and should not trigger challenge- stop in isolation (but may prompt extending the dosing interval). If peak flow is being assessed, then a decrease of ≥20% from baseline (assuming satisfactory technique) can be considered a stopping criteria |
| **Oral cavity symptoms** | Itchy mouth |  |  |  |
| **Throat/laryngeal symptoms** | Itchy throat, intermittent throat clearing | Persistent throat tightness or pain | Non-transient hoarseness, stridor | Subtle vocal changes are presumably due to mild laryngeal edema and should therefore trigger the FC to be stopped if non- transient in nature |
| **Abdominal discomfort** | Nausea (any severity)  Mild abdominal pain | Persistent non-distractable abdominal pain (usually with decrease in activity level in children)  Persistent severe abdominal pain |  | Abdominal pain is a subjective symptom and should not trigger challenge- stop in isolation. Persistent severe abdominal pain would normally be accompanied by other symptoms. Where this is present, further FC doses should be deferred to allow additional time for other symptoms to evolve |
| **Vomiting** | Vomit due to gag or taste aveersion |  | >1 episode of vomiting where investigator considers this due to allergic reaction | If vomiting occurs during or shortly after the FC dose, then this is more likely to be due to gag or taste aversion. If other symptoms subsequently develop, clinicians should reconsider whether the episode was non- allergic in origin |
| **Diarrhea** | One episode |  | >2 episode where investigator considers this due to allergic reaction |  |
| **Cardiovascular symptoms** | Mild tachycardia |  | Clinically significant hypotension or shock/collapse | Hypotension defined as a decrease in systolic BP greater than 30% from that person's baseline, OR (i) Under 10 years: sysBP  (ii) Under 10 years: sysBP <90 mmHg |
| **Neurological symptoms** | Feeling weak, tired, upset/agitated |  | Significant change in cognition or GCS (anaphylaxis) | Allergic mediators such as histamine are also neurotransmitters; neurological impairment can occur independently of cardiovascular compromise during allergic reactions |
