# Supplementary material for: Efficacy and safety of a 4-step versus a 5-step egg ladder in children with IgE-mediated hen's egg protein allergy: protocol for an open-label randomized controlled trial
Source: Front Allergy. 2025 Sep 15;6:1658186. doi: 10.3389/falgy.2025.1658186 (PMC12477030; doi:10.3389/falgy.2025.1658186)
Supplement: Supplementary file 2 [file Supplementaryfile1.docx]

**SUPPLEMENTARY MATERIAL 1. RECIPES FOR ORAL FOOD CHALLENGE BASED ON THE 5-STEP EGG LADDER**

1. **RECIPES FOR PARENTS**

| **1 STEP - MUFFIN (0,75 g of Hen's Egg Proteins (HEPs) per muffin)** Quantity: 8 muffins |
| --- |
| **INGREDIENTS**  DRY INGREDIENTS:  1 cup (125 g) all-purpose flour (wheat)  ½ cup (100 g) sugar  ¼ teaspoon (1.5 g) salt  1 teaspoon (5 g) baking powder  WET INGREDIENTS:  2 tablespoons (30 ml) canola oil  ½ teaspoon (2.5 ml) vanilla extract  1 large egg (50 g), beaten  ½ cup (120 ml) cow’s milk (if the child has no allergy to cow's milk proteins) or any calcium-fortified plant-based drink to which the child is not known to be allergic. |
| **INSTRUCTIONS**  1. Preheat oven to 180°C. This step may take 30-45 minutes. Bake muffins only in an oven that is completely preheated to 180°C.  2. Line a muffin pan with 8 muffin liners. Use aluminum or parchment paper muffin liners;  3. In a small mixing bowl, stir together the wet ingredients until well combined (canola oil, vanilla extract, egg, and milk or plant-based beverage). Set aside.  4. In a separate mixing bowl, mix together the dry ingredients (flour, sugar, salt, baking powder).  5. Add all wet ingredients to the dry ingredients at once and gently stir with a large spoon until wet and dry ingredients are combined. Do not stir for too long – small lumps may remain.  6. Divide the batter into the 8 prepared muffin liners. Try to make them similar in size, if possible.  7. Bake for 30 minutes or until golden brown and firm to the touch.  8. Let the muffins cool for 5 minutes in the tins before removing them to a wire rack. |

| **2 STEP - MUFFIN (1,5 g of Hen's Egg Proteins (HEPs) per muffin)** Quantity: 8 muffins |
| --- |
| **INGREDIENTS**  DRY INGREDIENTS:  1 cup (125 g) all-purpose flour (wheat)  ½ cup (100 g) sugar  ¼ teaspoon (1.5 g) salt  1 teaspoon (5 g) baking powder  WET INGREDIENTS:  2 tablespoons (30 ml) canola oil  ½ teaspoon (2.5 ml) vanilla extract  2 large eggs (50 g/per 1 egg), beaten  ½ cup (120 ml) cow’s milk (if the child has no allergy to cow's milk proteins) or any calcium-fortified plant-based drink to which the child is not known to be allergic. |
| **INSTRUCTIONS**  1. Preheat oven to 180°C. This step may take 30-45 minutes. Bake muffins only in an oven that is completely preheated to 180°C.  2. Line a muffin pan with 8 muffin liners. Use aluminum or parchment paper muffin liners;  3. In a small mixing bowl, stir together the wet ingredients until well combined (canola oil, vanilla extract, egg, and milk or plant-based beverage). Set aside.  4. In a separate mixing bowl, mix together the dry ingredients (flour, sugar, salt, baking powder).  5. Add all wet ingredients to the dry ingredients at once and gently stir with a large spoon until wet and dry ingredients are combined. Do not stir for too long – small lumps may remain.  6. Divide the batter into the 8 prepared muffin liners. Try to make them similar in size, if possible.  7. Bake for 30 minutes or until golden brown and firm to the touch.  8. Let the muffins cool for 5 minutes in the tins before removing them to a wire rack. |

| **3 STEP – PANCAKE**  Quantity: 6 pancakes Serving size for challenge: 1 pancake  Egg protein per serving: 2g |
| --- |
| **INGREDIENTS**  INGREDIENTS (for 6 pancakes)  2 eggs (50g/1 egg) pinch (0.5 g) salt 1 cup (125 g) all-purpose flour (wheat) 1/4 teaspoon (1.5 g) baking powder 200 ml cow’s milk (if the child has no allergy to cow's milk proteins) or any calcium-fortified plant-based drink to which the child is not known to be allergic. 50 ml carbonated mineral water 2 tablespoons (30 ml) canola oil |
| **INSTRUCTIONS:**  1. Whisk eggs and salt together in a bowl.  2. Gradually add the flour mixed with the baking powder, then the milk or plant-based drink, the canola oil, and finally the mineral water, stirring continuously to form a smooth dough.  3. Let the batter rest for 15 minutes.  4. Cook six equal-sized pancakes over medium heat for at least 2-3 minutes per side until golden brown. To get six equal-sized pancakes, divide the batter evenly into six cups and then bake these portions in the pan. |

1. **RECIPES FOR RESEARCHER**

For 4^th^ and 5^th^ steps egg will be cooked by a researcher.

| **4 STEP - HARD BOILED EGG**  Quantity: 2 eggs Serving size for challenge: 1 egg (50g) Egg protein per serving: 6g |
| --- |
| **INGREDIENTS**  2 eggs (50g/1 egg) water |
| **INSTRUCTIONS:**  1. Place the eggs in a pot and cover them with cold water. The water should cover the eggs by about 2 cm.  2. Put the pot on the stove and bring the water to a boil.  3. Once the water starts to boil, reduce the heat and cook the eggs for 10-12 minutes.  4. After 10-12 minutes, turn off the heat and pour cold water over the eggs.  5. Wait for the eggs to cool down, then peel them from their shells. |

| **5 STEP - SOFT BOILED EGG**  Quantity: 2 eggs Serving size for challenge: 1 egg (50g) Egg protein per serving: 6g |
| --- |
| **INGREDIENTS**  2 eggs (50g/1 egg) water |
| **INSTRUCTIONS:**  1. Place the eggs in a pot and cover them with cold water. The water should cover the eggs by about 2 cm.  2. Put the pot on the stove and bring the water to a boil.  3. Once the water starts to boil, reduce the heat and cook the eggs for 5 minutes.  4. After 5 minutes, turn off the heat and pour cold water over the eggs.  5. Wait for the eggs to cool down, then peel them from their shells. |
